# Chronic Low Back Pain and Incident Transient Ischemic Attack and Stroke in General Practices in Germany

**DOI:** 10.3390/healthcare11101499

**Published:** 2023-05-21

**Authors:** Louis Jacob, Lee Smith, Ai Koyanagi, Josep Maria Haro, Jae Il Shin, Christian Tanislav, Alexis Schnitzler, Karel Kostev

**Affiliations:** 1 Research and Development Unit, Parc Sanitari Sant Joan de Déu, CIBERSAM, ISCIII, Dr. Antoni Pujadas, 42, 08830 Barcelona, Spain; 2 Department of Physical Medicine and Rehabilitation, Lariboisière-Fernand Widal Hospital, AP-HP, University Paris Cité, 75010 Paris, France; 3 Epidemiology of Ageing and Neurodegenerative Diseases, Université Paris Cité, Inserm U1153, 75010 Paris, France; 4 Centre for Health, Performance and Wellbeing, Anglia Ruskin University, Cambridge CB1 1PT, UK; 5 Institució Catalana de Recerca i Estudis Avançats (ICREA), Pg. Lluis Companys 23, 08010 Barcelona, Spain; 6 Department of Pediatrics, Yonsei University College of Medicine, Seoul 03722, Republic of Korea; 7 Department of Geriatrics and Neurology, Diakonie Hospital Jung Stilling Siegen, 57074 Siegen, Germany; christian.tanislav@diakonie-sw.de; 8 Epidemiology, IQVIA, 60549 Frankfurt, Germany; 9 Department of Gynecology and Obstetrics, University Clinic of Marburg, 35043 Marburg, Germany

**Keywords:** chronic low back pain, transient ischemic attack, stroke, Germany, retrospective cohort study, epidemiology

## Abstract

The aim was to investigate the association between chronic low back pain (CLBP) and incident transient ischemic attack (TIA) and stroke in Germany. The present retrospective cohort study included adults aged ≥18 years who were diagnosed for the first time with CLBP in one of 1198 general practices in Germany in 2005–2019 (index date). Patients without CLBP were matched to those with CLBP (1:1) using a propensity score based on age, sex, the index year, the number of medical consultations per year during the follow-up, and the number of years of follow-up. In patients without CLBP, the index date was a randomly selected visit date. Both groups were followed for up to 10 years. There were 159,440 patients included in the study (mean (SD) age: 52.1 (16.5) years; 51.5% women). Within 10 years of the index date, 6.5% and 5.9% of patients with and without CLBP were diagnosed with TIA or stroke, respectively (log-rank *p*-value < 0.001). The Cox regression analysis corroborated these results, as there was a significant association between CLBP and incident TIA or stroke (HR = 1.28, 95% CI = 1.22–1.35). CLBP was positively and significantly associated with incident TIA and stroke in Germany. More research is warranted to better understand this relationship.

## 1. Introduction

Stroke is a neurological disorder with a vascular origin characterized by neurological disturbance lasting for more than 24 h or resulting in death [1]. The two major types of stroke are hemorrhagic and ischemic strokes. Transient ischemic attack (TIA), a risk factor for subsequent stroke, is a condition defined by temporary neurological dysfunction without acute infarction and is caused by ischemia of the brain, spinal cord, or retina [1]. In 2019, around 101 million people had a history of stroke, and approximately 12.2 million individuals were newly diagnosed with stroke in the world [2]. Stroke has been identified as the second leading cause of death and the third leading cause of death or disability worldwide, and this neurological disorder is associated with a major economic burden at the international level [3]. Given that the global population is aging, the burden associated with stroke is steadily increasing. Taking these data together, it is of utmost importance to better identify risk factors for and protective factors against TIA and stroke.

One potential understudied risk factor for these two neurological diseases is chronic low back pain (CLBP). CLBP is defined as pain in the lower back persisting for over three months [4]. CLBP is one of the most frequent musculoskeletal disorders, and the prevalence of CLBP is particularly high in middle-aged adults and women [5]. To the best of the authors’ knowledge, to date, only one retrospective cohort study from Taiwan has analyzed the association between CLBP and stroke, and it found a positive and significant relationship [6]. Several factors may explain the CLBP–stroke association, such as insufficient physical activity [7,8], several physical (e.g., diabetes [9,10] and coronary heart disease [11,12]) and psychiatric (e.g., anxiety [13,14] and depression [13,15]) conditions, the long-term use of multiple drugs (e.g., non-steroidal anti-inflammatory drugs [16,17] and antidepressants [17,18]), and adverse life events (e.g., divorce [19,20] and job loss [21,22]). Although the study mentioned above has advanced the field, this study has limitations that need to be acknowledged. First, the study focused on stroke alone, and there is no data on the potential effects of CLBP on TIA. Second, no distinction was made between CLBP with and without lumbosacral radiculopathy, although there are substantial differences in the epidemiology, treatment, and management of these two conditions [23,24]. Third, data were collected in Taiwan, and the findings may not be extrapolated to other countries. More research is warranted on the potential relationship between CLBP, TIA, and stroke in this context.

Therefore, the purpose of this retrospective cohort study was to investigate the relationship between CLBP and incident TIA and stroke in the German population, using data from a large sample of adults followed in general practices. The hypothesis was that people with CLBP would be at an increased odds for TIA and stroke compared with those without stroke. Pending that the hypothesis of the study is corroborated, the findings would highlight the importance of the early treatment and management of CLBP in primary care.

## 2. Methods

### 2.1. Ethics Approval and Consent to Participate

Based on German law, anonymous electronic medical data can be used for research, provided certain conditions are met. This legislation allows the use of these deidentified records without obtaining written informed consent from the patients and approval from a medical ethics committee.

### 2.2. Database

The present retrospective cohort study used data from the Disease Analyzer database (IQVIA). This database has been described previously [25]. Briefly, the Disease Analyzer database contains demographic, diagnosis, and prescription data anonymously, which are regularly obtained from the computer systems of general and specialized practices in Germany. Diagnoses are coded using the International Classification of Diseases, 10th revision (ICD-10), while prescriptions are coded using the Anatomical Classification of Pharmaceutical Products of the European Pharmaceutical Market Research Association (EphMRA). Data quality is assessed every month using several criteria (e.g., completeness of documentation and linkage between diagnoses and prescriptions). Practices included in the Disease Analyzer database are selected based on information (i.e., physician’s age, specialty group, community size category, and German federal state) published on a yearly basis by the German Medical Association. Finally, the database includes approximately 3% of all practices in Germany, and this panel of practices has been found to be representative of the country [25].

### 2.3. Population

**Figure 1:**
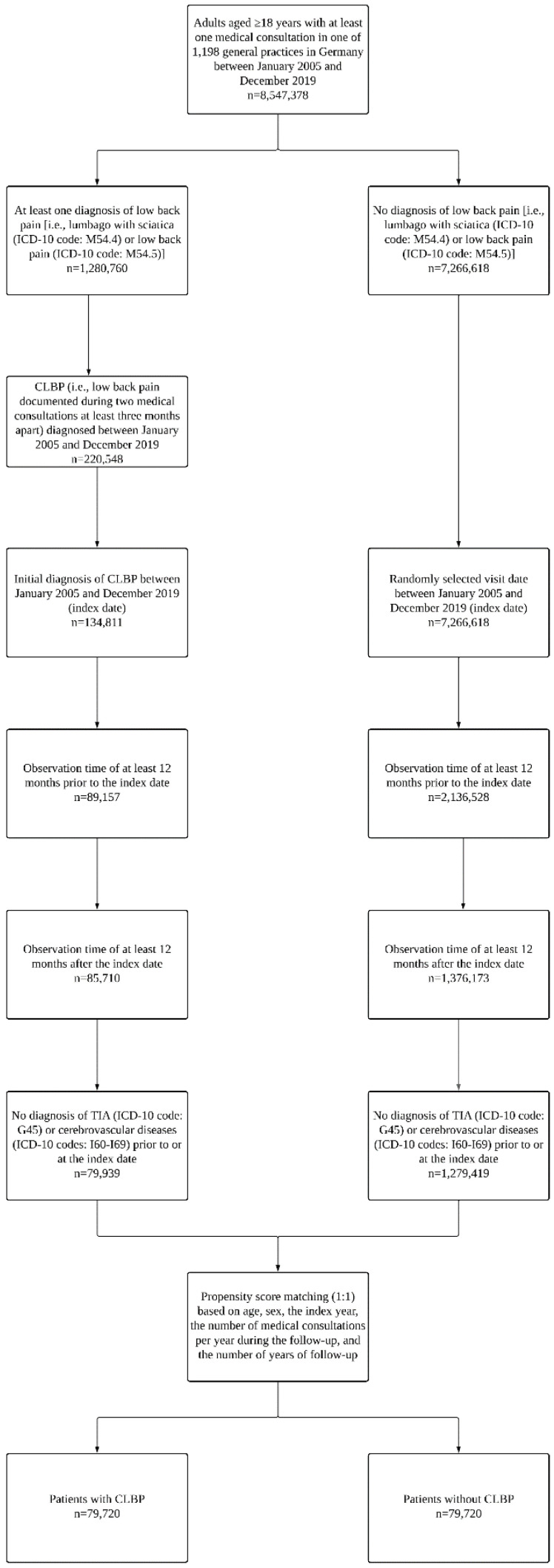
Flow chart of the study participants. Abbreviations: ICD-10, International Classification of Diseases, 10th revision; CLBP, chronic low back pain; TIA, transient ischemic attack. Low back pain corresponded to the presence of at least one of the following two ICD-10 codes: M54.4 (i.e., lumbago with sciatica) and M54.5 (i.e., low back pain).

This study included adults aged ≥18 years who were diagnosed for the first time with CLBP in one of 1,198 general practices in Germany between January 2005 and December 2019 (index date). CLBP corresponded to low back pain (i.e., lumbago with sciatica (ICD-10 code: M54.4) or low back pain (ICD-10 code: M54.5)) documented during two different medical consultations at least three months apart. The use of the ICD-10 codes M54.4 and M54.5 to define low back pain was based on prior literature [26,27,28]. Additional inclusion criteria were the following: observation time of at least 12 months prior to the index date; observation time of at least 12 months after the index date; no diagnosis of TIA (ICD-10 code: G45) or cerebrovascular diseases (ICD-10 codes: I60-I69) prior to or at the index date. After applying similar inclusion criteria, individuals without CLBP were matched (1:1) to those with CLBP using a propensity score based on age, sex, the index year, the number of medical consultations per year during the follow-up, and the number of years of follow-up. For adults without CLBP, the index date corresponded to a randomly selected visit date between January 2005 and December 2019. The flow chart of the study participants is displayed in Figure 1.

### 2.4. Outcome

The outcome of this study was the incidence of TIA (ICD-10 code: G45) and stroke (ICD-10 codes: I60-I64) in the 10 years following the index date in the group with and without CLBP. Stroke included hemorrhagic (ICD-10 codes: I60-I62), ischemic (ICD-10 code: I63), and unspecified stroke (ICD-10 code: I64). The end of follow-up corresponded to the diagnosis of TIA or stroke or the last medical consultation in the 10 years following the index date.

### 2.5. Covariates

We selected covariates based on previous literature [6], and these covariates included age, sex, the number of medical consultations per year during the follow-up, the number of years of follow-up, several chronic physical and psychiatric conditions diagnosed prior to the end of follow-up, and several drugs prescribed prior to the end of follow-up. Chronic physical and psychiatric conditions were essential hypertension (ICD-10 code: I10), disorders of lipoprotein metabolism and other lipidemias (ICD-10 code: E78), osteoarthritis (ICD-10 codes: M15–M19), depression (ICD-10 codes: F32 and F33), diabetes mellitus (ICD-10 codes: E10–E14), ischemic heart diseases (ICD-10 codes: I20–I25), overweight and obesity (ICD-10 code: E66), anxiety disorders (ICD-10 code: F41), cancer (ICD-10 codes: C00–C97), chronic kidney disease and unspecified kidney failure (ICD-10 codes: N18 and N19), and atrial fibrillation and flutter (ICD-10 code: I48). Drugs included nonsteroidal anti-inflammatory drugs (EphMRA code: M01A), antidepressants (EphMRA code: N06A), and antipsychotics (EphMRA code: N05A). These chronic physical and psychiatric conditions and drugs have been found to be positively associated with TIA and stroke.

### 2.6. Statistical Analyses

Characteristics were compared between patients with and without CLBP using McNemar tests for categorical variables with two categories, Stuart–Maxwell tests for categorical variables with more than two categories, and Wilcoxon signed-rank tests for continuous variables. The 10-year incidence of TIA or stroke in the group with and without CLBP was further studied using Kaplan–Meier curves and log-rank tests. Analyses were repeated for TIA and each type of stroke (i.e., hemorrhagic, ischemic, and unspecified) separately. The association between CLBP (independent variable) and incident TIA or stroke (dependent variable) in the overall population and age (i.e., ≤40, 41–50, 51–60, 61–70, and >70 years) and sex subgroups (i.e., female and male) was investigated using Cox regression models adjusted for the chronic physical and psychiatric conditions and prescribed drugs previously mentioned. Sensitivity analyses were conducted by type of neurological disorder (i.e., TIA and hemorrhagic, ischemic, and unspecified stroke) and by type of CLBP (i.e., lumbago with sciatica and low back pain). The results of the Cox regression analyses are presented as hazard ratios (HRs) and 95% confidence intervals (CIs). *p*-values < 0.050 were considered statistically significant. All analyses were performed using SAS 9.4 (SAS Institute, Cary, USA).

## 3. Results

**Figure 2:**
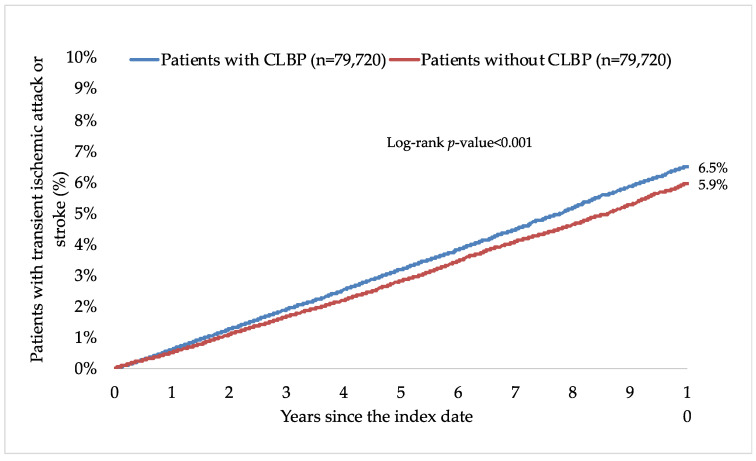
Ten-year cumulative incidence of transient ischemic attack or stroke in people with and without CLBP (Kaplan–Meier curves). Abbreviation: CLBP, chronic low back pain.

**Figure 3:**
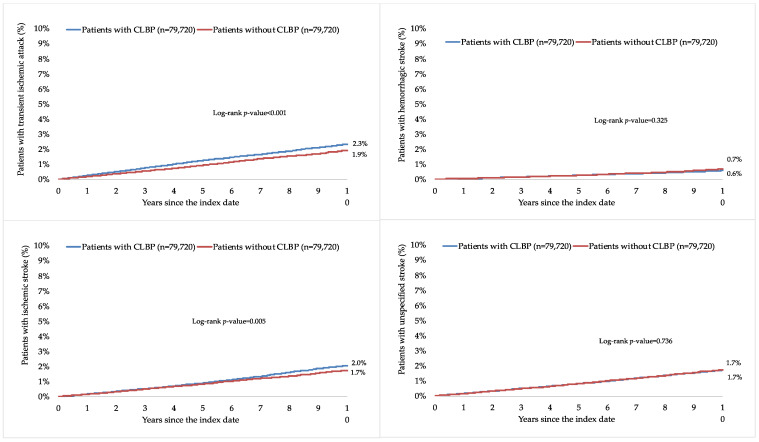
Ten-year cumulative incidence of transient ischemic attack, hemorrhagic stroke, ischemic stroke, and unspecified stroke in people with and without CLBP (Kaplan–Meier curves). Abbreviation: CLBP, chronic low back pain.

This retrospective cohort study included 79,720 patients with and 79,720 patients without CLBP. The characteristics of the study participants after 1:1 matching are displayed in Table 1. The mean (standard deviation) age at the index date was 52.1 (16.5) years in both groups, and the prevalence of women was 51.5%. The mean (standard deviation) number of medical consultations per year during the follow-up was 8.7 (3.4) in the group with CLBP and 8.6 (3.4) in the group without CLBP. The participants were followed up for 6.6 (3.9) years (mean (standard deviation)). The three most frequent chronic comorbidities were essential hypertension (56.0% in those with and 57.2% in those without CLBP), disorders of lipoprotein metabolism and other lipidemias (39.4% and 39.4%), and osteoarthritis (38.7% and 29.2%). Within 10 years of the index date, 6.5% and 5.9% of patients with and without CLBP were diagnosed with TIA and stroke, respectively (log-rank *p*-value < 0.001; Figure 2). The respective figures were 2.3% and 1.9% for TIA (log-rank *p*-value < 0.001), 0.6% and 0.7% for hemorrhagic stroke (log-rank *p*-value = 0.325), 2.0% and 1.7% for ischemic stroke (log-rank *p*-value = 0.005), and 1.7% and 1.7% for unspecified stroke (log-rank *p*-value = 0.736; Figure 3). Table 2 shows the results of the adjusted Cox regression analyses. After adjusting for the chronic physical and psychiatric conditions (i.e., essential hypertension, disorders of lipoprotein metabolism and other lipidemias, osteoarthritis, depression, diabetes mellitus, ischemic heart diseases, overweight and obesity, anxiety disorders, cancer, chronic kidney disease and unspecified kidney failure, and atrial fibrillation and flutter) and drugs (i.e., nonsteroidal anti-inflammatory drugs, antidepressants, and antipsychotics), there was a positive and significant association between CLBP and incident TIA or stroke (HR=1.28, 95% CI = 1.22–1.35). These findings were corroborated in all age and sex subgroups except in people aged ≤40 years, with HR ranging from 1.24 (95% CI = 1.07–1.43) in those aged 41–50 years to 1.34 (95% CI = 1.23–1.45) in those aged >70 years. Furthermore, a significant relationship was observed between CLBP and the incidence of TIA (HR = 1.42, 95% CI = 1.30–1.55), ischemic stroke (HR = 1.32, 95% CI = 1.20–1.45), and unspecified stroke (HR = 1.16, 95% CI = 1.05–1.28) but not between CLBP and hemorrhagic stroke (HR = 1.03, 95% CI = 0.87–1.22). Finally, both lumbago with sciatica (HR = 1.27, 95% CI = 1.18–1.38) and low back pain (HR = 1.28, 95% CI = 1.20–1.37) were significantly associated with incident TIA or stroke.

## 4. Discussion

### 4.1. Main Findings

In the present retrospective cohort study of more than 159,000 adults from approximately 1200 general practices in Germany, the 10-year incidence of TIA and stroke was 6.5% and 5.9% in those with and without CLBP (log-rank *p*-value < 0.001), respectively. Similar findings were obtained in the adjusted Cox regression analyses, and CLBP was associated with a significant 1.28-fold increase in the risk of TIA or stroke. In terms of individual neurological conditions, the relationship was significant for TIA, ischemic stroke, and unspecified stroke. Finally, both lumbago with sciatica and low back pain significantly predicted the incidence of TIA and stroke. To the best of the authors’ knowledge, this is only the second study investigating the relationship between CLBP, TIA, and stroke, while it is the largest study on the topic to date and the only study from Germany.

### 4.2. Interpretation of Findings

The present findings align with the results of the sole study in which the association between CLBP and stroke has been previously analyzed. In this nationwide population-based cohort of 30,924 individuals in Taiwan, those with CLBP were more likely to be diagnosed with stroke than those without CLBP (HR = 2.35, 95% CI = 2.14–2.57), and the association was more pronounced for ischemic (HR=2.41, 95% CI=2.18–2.66) than for hemorrhagic stroke (HR = 1.55, 95% CI = 1.16–2.06) [6]. Interestingly, HRs obtained in the present study were lower than those obtained in the previous study, and CLBP was not significantly associated with hemorrhagic stroke. Two major differences between these research bodies can explain the discrepancies in the study findings. First, data from these two cohorts were collected in Germany and Taiwan, and the treatment and management of CLBP and the epidemiology of stroke may differ between these countries. Second, the study from Taiwan did not include several conditions (e.g., overweight and obesity [29], osteoarthritis [30], and anxiety disorders [14]) and drugs known to be associated with stroke (e.g., antidepressants [18] and antipsychotics [31]), potentially leading to an overestimation of the effect size of the CLBP–stroke relationship. The present study conducted in Germany not only partially corroborates the previous findings mentioned above but also shows that the association is significant in most age and sex groups and exists for both lumbago with sciatica and low back pain. The lack of a significant association observed for hemorrhagic stroke is critical and highlights substantial differences in risk factors for hemorrhagic and ischemic stroke [32,33]. Finally, this study adds to the literature by identifying a positive and significant association between CLBP and TIA, and this result strengthens the evidence on the effects of CLBP on cerebrovascular diseases.

There are several hypotheses to explain the relationship between CLBP, TIA, and stroke. Inflammation and inflammatory biomarkers (e.g., C-reactive protein (CRP), tumor necrosis factors, and interleukin 6) likely play a role in the chronicization of low back pain, potentially via the prolonged activation of peripheral nociceptors, hyperalgesia, and stress. A systematic review of 13 studies indicated that levels of CRP and tumor necrosis factors were significantly higher in people with low back pain than those without low back pain [34]. Meanwhile, inflammation is a risk factor for ischemic stroke, and the association is mediated by thrombogenesis, platelet activation, and atherosclerosis [35]. A study of 30,239 adults aged ≥45 years from the United States showed that high CRP was positively and significantly associated with incident ischemic stroke [36]. Interestingly, some preliminary data also suggest that inflammatory biomarkers are increased in patients with TIA compared with those without TIA [37]. In addition, although the statistical analyses were adjusted for several chronic physical and psychiatric conditions, other disorders may be involved in the relationship between CLBP and incident TIA and stroke. For example, CLBP is a frequent symptom in osteomyelitis and inflammatory bowel diseases [38,39], and osteomyelitis and inflammatory bowel diseases are risk factors for TIA and stroke [40,41]. Moreover, adults with CLBP are less likely to be physically active than their counterparts without CLBP, and pain and disability may favor the lack of physical activity in the CLBP population [8]. In a sample of 3,220 adults with CLBP living in Spain, the prevalence of low physical activity was 30%, and several factors were significantly associated with low physical activity (e.g., female sex, obesity, and the inability to walk 500 m) [42]. In turn, insufficient physical activity positively predicts the occurrence of TIA [43] and stroke [7], potentially via an increased risk for hypertension, diabetes, and dyslipidemia. Finally, people with CLBP display higher odds of divorce [19] and job loss [22] compared with the general population, likely owing to the fact that chronic pain interferes with personal and professional daily life. Interestingly, divorce [20] and job loss [21] are positively associated with incident stroke, and this relationship may be mediated by poor mental health (e.g., high levels of stress, anxiety, and depression) and engaging in unhealthy behaviors (e.g., fast food consumption, smoking, and alcohol misuse).

### 4.3. Clinical Implications and Directions for Future Research

Based on the findings of this study, adults with CLBP are at an increased risk for TIA and stroke compared with those without CLBP. Given the high prevalence of CLBP worldwide, primary care plays a major role in the treatment and management of this condition [24]. To prevent the occurrence of CLBP, patients with acute low back pain should be reassured about the favorable prognosis of lumbago and advised to continue their activities of daily living. The management of CLBP is multidisciplinary and involves, for example, exercise therapy, the prescription of antidepressants to alleviate pain and anxiety and depressive symptoms, and psychosocial interventions. Exercise therapy is the core treatment for CLBP and may include strengthening and stretching exercises at home or in a group. General practitioners may also aim at the prevention and treatment of the frequent physical comorbidities of CLBP, which can further increase the risk of TIA and stroke (e.g., diabetes, hypertension, and obesity). Finally, in terms of future research, more studies are needed to corroborate or invalidate these results in other countries and settings. More data are also warranted to better understand the mediating factors involved in the association between CLBP, TIA, and stroke.

### 4.4. Strengths and Limitations

The strengths of this study are its large sample size, the duration of follow-up, and the use of data obtained in primary care practices. However, the study findings should be interpreted in light of several limitations. First, the diagnosis of CLBP relied on ICD-10 codes only, and there were no data on the severity of symptoms. Thus, we could not investigate if the risk of TIA and stroke increased with the severity of CLBP symptoms. Second, patients with low back pain were those with lumbago with sciatica and those with low back pain without sciatica. Although this definition was based on prior literature [26,27,28], the physiopathological mechanisms of pain may differ in the presence and absence of sciatica, potentially resulting in a heterogeneous population. Third, for most patients with TIA and stroke, it is likely that the diagnosis has been made in other settings (e.g., at the hospital or in neurological practices), and these neurological conditions may have been underdocumented in general practices. Fourth, there were no data on health behaviors (e.g., physical activity, smoking habit, and alcohol consumption), and this lack of data may have introduced biases in the statistical analyses. Fifth, given that the treatment and management of CLBP likely vary at the international level, these findings obtained in Germany may not be generalized to other countries.

## 5. Conclusions

In this retrospective cohort study, including 159,000 adults from approximately 1200 general practices in Germany, CLBP was positively and significantly associated with incident TIA and stroke. Regarding individual neurological disorders, the relationship was significant for TIA, ischemic stroke, and unspecified stroke but not hemorrhagic stroke. Finally, these results should be corroborated in other countries and settings, and future research may focus on the mediators involved in these associations.

## Figures and Tables

**Table 1:** Characteristics of study patients after 1:1 matching.

Variable	Patients with CLBP (*n* = 79,720)	Patients without CLBP (*n* = 79,720)	*p*-Value *
Age at the index date (in years)			
Mean (standard deviation)	52.1 (16.5)	52.1 (16.5)	0.632
≤40	25.2	25.1	0.066
41–50	21.4	21.4	
51–60	23.1	22.7	
61–70	14.1	14.6	
>70	16.2	16.2	
Sex			
Female	51.5	51.5	1.000
Male	48.5	48.5	
Number of medical consultations per year during the follow-up, mean (standard deviation)	8.7 (3.4)	8.6 (3.4)	0.064
Number of years of follow-up, mean (standard deviation)	6.6 (3.9)	6.6 (3.9)	1.000
Chronic physical and psychiatric conditions diagnosed prior to the end of follow-up ^†^			
Essential hypertension	56.0	57.2	<0.001
Disorders of lipoprotein metabolism and other lipidemias	39.4	39.4	0.782
Osteoarthritis	38.7	29.2	<0.001
Depression	36.2	31.5	<0.001
Diabetes mellitus	22.7	24.9	<0.001
Ischemic heart diseases	19.4	19.1	0.162
Overweight and obesity	18.6	16.9	<0.001
Anxiety disorders	14.6	12.7	<0.001
Cancer	13.9	14.5	<0.001
Chronic kidney disease and unspecified kidney failure	8.6	8.7	0.281
Atrial fibrillation and flutter	8.4	9.3	<0.001
Drugs prescribed prior to the end of follow-up			
Nonsteroidal anti-inflammatory drugs	89.3	65.0	<0.001
Antidepressants	30.6	26.3	<0.001
Antipsychotics	9.6	9.3	0.046

Abbreviation: CLBP, chronic low back pain. Data are percentages unless otherwise specified. * *p*-values were obtained using McNemar tests for categorical variables with two categories, Stuart–Maxwell tests for categorical variables with more than two categories, and Wilcoxon signed-rank tests for continuous variables. ^†^ The end of follow-up corresponded to the diagnosis of transient ischemic attack or stroke or the last medical consultation in the 10 years following the index date.

**Table 2:** Associations between CLBP and incident TIA and stroke in general practices in Germany (adjusted Cox regression analyses).

	Incidence in Patients with CLBP *	Incidence in Patients without CLBP *	HR (95% CI)	*p*-Value
TIA and stroke ^†^				
Overall population	6.9	6.1	1.28 (1.22–1.35)	<0.001
Age ≤40 years	1.3	1.4	0.96 (0.76–1.20)	0.687
Age 41–50 years	3.7	3.3	1.24 (1.07–1.43)	0.005
Age 51–60 years	6.0	5.5	1.26 (1.13–1.42)	<0.001
Age 61–70 years	10.9	9.6	1.29 (1.16–1.43)	<0.001
Age >70 years	19.1	16.2	1.34 (1.23–1.45)	<0.001
Female sex	6.3	6.2	1.25 (1.17–1.34)	<0.001
Male sex	7.4	6.0	1.31 (1.22–1.42)	<0.001
By type of TIA and stroke ^‡^				
TIA	2.5	1.9	1.42 (1.30–1.55)	<0.001
Hemorrhagic stroke	0.6	0.6	1.03 (0.87–1.22)	0.746
Ischemic stroke	2.1	1.8	1.32 (1.20–1.45)	<0.001
Unspecified stroke	1.8	1.7	1.16 (1.05–1.28)	0.003
By type of low back pain ^§^				
Lumbago with sciatica	7.2	6.5	1.27 (1.18–1.38)	<0.001
Low back pain	6.6	5.8	1.28 (1.20–1.37)	<0.001

Abbreviations: CLBP, chronic low back pain; TIA, transient ischemic attack; HR, hazard ratio; CI, confidence interval. Cox regression analyses were adjusted for chronic physical and psychiatric conditions and drugs documented prior to the end of follow-up. The end of follow-up corresponded to the diagnosis of TIA or stroke or the last medical consultation in the 10 years following the index date. * The incidence was defined as the number of cases per 1,000 person-years. ^†^ In these analyses conducted in the overall population and age and sex subgroups, TIA and stroke were studied as a single dependent variable. ^‡^ In these analyses conducted in the overall population, TIA and stroke were studied as separate dependent variables. ^§^ In these analyses conducted in the overall population, lumbago with sciatica and low back pain were studied as separate independent variables.

## Data Availability

The data and the code used for this study are available from the corresponding author upon reasonable request.
